# DiGeorge Syndrome Presenting as Hypocalcaemia-Induced Seizures in Adulthood

**DOI:** 10.1155/2013/923129

**Published:** 2013-05-20

**Authors:** Adrian Zammit, Deborah Grech Marguerat, Josephine Psaila, Alexander Attard

**Affiliations:** Department of Surgery, Mater Dei Hospital, Msida MSD 2090, Malta

## Abstract

*Introduction*. DiGeorge syndrome is a developmental defect commonly caused by a microdeletion on the long arm of chromosome 22 or less frequently by a deletion of the short arm of chromosome 10. *Case report*. We report a case of a gentleman with mild dysmorphic features who presented with hypocalcaemia-induced seizures and an associated thyroid mass with a background of learning difficulties and abnormal immune function. *Discussion*. DiGeorge syndrome was initially described in 1967 by Angelo DiGeorge. The majority of cases are due to a novel mutation. The resulting learning difficulties, congenital heart disease, palatal abnormalities, hypoplasia/aplasia of the parathyroid and thymus glands, and immune deficiency generally lead to diagnosis in childhood. Presentation in adulthood is rare but must be borne in mind when dealing with cases of hypocalcaemia even in the absence of florid phenotypic features. A link with malignant disease has also been reported and should lead to prompt investigation of concerning masses.

## 1. Introduction

DiGeorge syndrome is a relatively common developmental defect, due to a microdeletion of chromosome 22q11.2, and less commonly 10p13. This is associated with developmental defects, including hypoplasia of the thymus and parathyroid glands, facial dysmorphism, congenital heart disease, palatal abnormalities and renal malformations, as well as learning difficulties. We report a rare case of DiGeorge syndrome presenting in adulthood as hypocalcaemia-induced seizures and a thyroid mass.

## 2. Case Report

A 34-year-old gentleman with mild dysmorphic features presented to the emergency department with a sudden onset loss of consciousness which lasted approximately 1 minute, during which he sustained tonic-clonic movements which self-terminated. A 1 year history of episodic carpopedal spasms and upper limb paraesthesia was elicited. A head computed tomography (CT) scan was normal. A corrected calcium level was 1.17 mmol/L.

His past medical history included a febrile seizure at 2 years of age, with no history suggestive of absence seizures or myoclonic jerks. He was operated for testicular torsion, acute appendicitis, a congenital epigastric hernia, a supraumbilical hernia, and a removal of an accessory digit in the left hand, the latter of which was also present in relatives from his paternal side. His developmental history included gestation at term with a birth weight of 3.2 kg and a delayed growth spurt. Until the age of 13 years he had regular physiotherapy for recurrent chest and sinus infections, with multiple hospital admissions. At that point he was thoroughly investigated and a diagnosis of cystic fibrosis was suspected, with equivocal sodium sweat tests. Heaf tuberculin skin test (Sterneedle test) in 1985, in spite of previous TB vaccination, was negative. During his adolescence he also reported incapacitating muscle cramps, which later resolved. Learning difficulties were attributed to his frequent absence from school, but the patient was now in full time employment.

On examination there was mild facial dysmorphism with a short philtrum. He was haemodynamically stable, with an early diastolic murmur. Chest was clear. Examination of the neck revealed a grossly enlarged right thyroid lobe, with deviation of the trachea to the left. 

**Figure 1:**
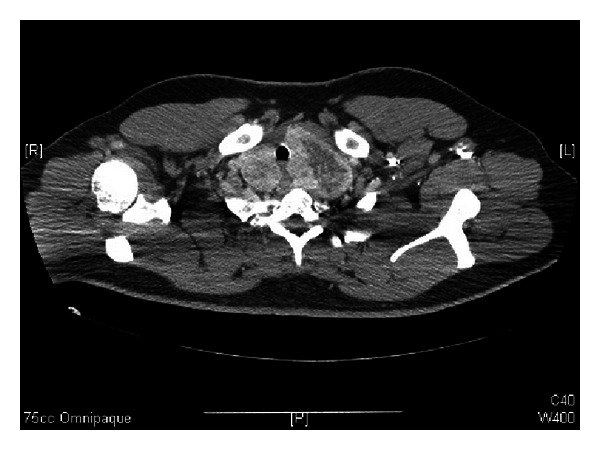
Axial CT scan with contrast at the level of T2, showing an enlarged thyroid gland, larger on the left, with tracheal deviation and inhomogeneous enhancement.

**Figure 2:**
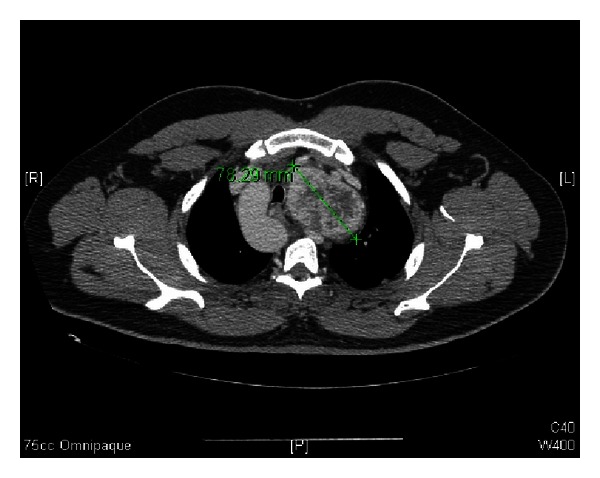
Axial CT scan with contrast at T4, showing retrosternal extension of the left thyroid lobe, with maximal diameter of 78 mm.

**Figure 3:**
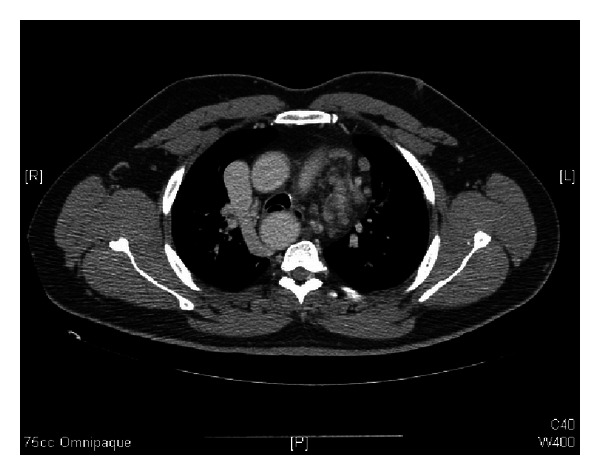
Axial CT scan at T5 revealing the presence of an incidental right-sided thoracic aorta.

Thyroid function tests and antithyroid antibodies were normal. Serum parathyroid hormone levels were low. Serum calcitonin levels were elevated. Other routine blood investigations, including magnesium levels, were normal. A CT scan of the neck and thorax showed a grossly enlarged, inhomogeneous thyroid gland which was extending deep into the upper mediastinum and associated with enlarged cervical lymph nodes (Figures 1 and 2). An incidental right-sided aortic arch was also noted (Figure 3). In view of the malignant features on CT, a total thyroidectomy and lymphadenectomy was performed. Histopathology reported the presence of two separate follicular adenomata and a nodular goitre.

The patient was discharged on calcium supplements, alpha-calcidol and antiepileptic drugs. The latter were initiated in view of tonic carpo-pedal spasms, which were initially considered to be suggestive of partial seizure activity. A brain magnetic resonance (MR) scan was unremarkable. No further seizures were reported during the follow-up period with continuation of calcium supplementation and the antiepileptic treatment was stopped.

Fluorescence in situ hybridisation (FISH) analysis later confirmed the diagnosis of DiGeorge syndrome with a deletion on the long arm of chromosome 22-22q11.2 deletion.

## 3. Discussion

DiGeorge syndrome, which was originally described in 1967 by Di George et al. [1], is associated with microdeletions of chromosome 22q11.2 and less commonly chromosome 10p13. It has an estimated prevalence ranging from 1 in 4000 to 1 in 6395 [2, 3]. In view of the variable penetrance, the incidence is probably much higher. Most cases (93%) have a *de novo* deletion, whereas the remaining 7% have inherited the deletion. Inheritance is in an autosomal dominant manner. Familial cases have also been described.

The syndrome is associated with failure of development of the third and fourth branchial pouches [4]. Mølsted et al. [5] suggested a failure of or aberrant migration of the neural crest during the fourth week of embryogenesis. The defective migration of these cells leads to the syndrome which is associated with variable findings that are, as exemplified by our case, not always clinically pronounced. These include congenital heart diseases (74%), palatal abnormalities (69%), learning difficulties (80%) [6], hypoplasia or aplasia of the parathyroid glands and thymus glands, which cause hypocalcaemia (50%) and immune deficiency (70%) [7]. Behavioural and psychiatric morbidity is also prevalent, with higher rates of attention deficit hyperactivity disorder (ADHD), affective disorder, schizophrenia and anxiety than the normal population, particularly in adults [8]. In an observational study, 30% of adults with deletions of chromosome 22q11 experienced a psychotic disorder, with approximately 25% of subjects satisfying the diagnostic criteria for schizophrenia [9]. Less frequent findings which have been reported include hearing loss, growth hormone deficiency, autoimmune disorders, and increased incidence of unprovoked seizures in the absence of hypocalcaemia [10]. Typical dysmorphic features for this syndrome have been described. However these may be subtle, particularly in cases diagnosed in adulthood, in whom these may be absent. Characteristic dysmorphic features include micrognathia, short philtrum with fish-mouth appearance, antiamygdaloid slant of the lateral chantus, telecanthus with short palpebral fissures, hypertelorism, and low-set ears [11, 12].

Congenital heart defects are the major cause of mortality in this syndrome and have been reported in 75% of patients [12]. Findings include tetralogy of Fallot, aortic arch anomalies and ventricular and atrial septal defects. 

Associated palatal abnormalities may not be a presenting feature, although patients with this syndrome often have unrecognised abnormalities. The commonest abnormality is velopharyngeal incompetence. Only about 17% of patients have no palatal involvement.

Other systems are also involved. Skeletal findings include polydactyly of the hands, accessory ribs, hemivertebrae, and craniosynostosis. Renal anomalies, laryngotracheal abnormalities, ophthalmological findings and CNS involvement have been reported. Gastrointestinal anomalies have been found in association with this syndrome, including umbilical and diaphragmatic hernias, accessory spleens, oesophageal and jejunal atresia, and Hirschsprung's disease.

Immune system involvement is secondary to abnormal development of the thymus, with subsequent aberrant development of T cells [13]. In the majority of patients, this T cell immunodeficiency is surprisingly milder than expected. In this latter group, usually classified as “partial” DiGeorge syndrome, there is a lower number of functional circulating T cells. There is evidence that in this group of patients further development of T cells occurs within the first years of life, with both an increase in production of T cells through homeostatic expansion and further immune function development [14]. Only a few studies investigating the immunological function of adult DiGeorge patients exist, however these indicate a trend towards normal T cell counts in adulthood [15]. Opportunistic infections in adulthood are therefore rarely reported [16]. However, as homeostatic expansion leads to T cells with shorter telomeres that in turn leads to premature senescence and lower T cell recombination [17, 18], the effectiveness of T cell function has been hypothesized to decline with age. This could explain the lack of a positive response to the Heaf tuberculin skin test in a previously immunised patient, as seen in our case. Humoral immune responses are also affected. These include evidence of IgA deficiency, impaired responses to vaccines, and reports of hypogammaglobulinaemia, with specific antibody deficiencies being described in over half the DiGeorge subjects in one study [19–21].

The T cell immunodeficiency is of major significance in patients with “complete” DiGeorge syndrome and aplasia of the thymus, where affected patients need immune reconstitution, usually with bone marrow transplantation or thymic transplants. This is fortunately rare, occurring in less than 1% of reported cases [22]. The disruption of the thymic function also predisposes these patients to autoimmune diseases, with homeostatic expansion showing a tendency for self-reactive T cells and a decrease in regulatory T cells [23, 24]. 

A number of reports have also associated DiGeorge syndrome with an increased risk of malignancy [25]. This could be accounted for by the patient's immunodeficiency, chronic inflammation secondary to recurrent infections, and deletion of cathecol-O-methyltransferase (COMT) gene which is usually included in the hemizygous deletion, the product of which is involved in toxin metabolism. Lymphomas, leukaemias, neuroblastomas, hepatoblastomas, Wilm's tumour, renal cell carcinoma, and thyroid carcinoma have all been reported in patients with DiGeorge syndrome. All were age appropriate, apart from the occurrence of thyroid carcinoma in a young patient. 

Age of presentation depends on the severity and type of defects associated with the condition. Very early presentation in the neonatal period is usually due to presentation with cardiac disease or severe hypocalcaemia. Patients with minimal facial features, recurrent infections, or mild cardiac disorders are diagnosed later on in childhood.

## 4. Conclusion

This is a rare case of DiGeorge syndrome, who presented in adulthood with hypocalcaemia-induced seizures. Few cases of adult presentation have been described in the literature [26, 27]. The patient had extensive but nonspecific complaints throughout his childhood, including delayed development of immune function, skeletal findings, learning difficulties, and cardiac abnormalities. Although rare, DiGeorge syndrome should be considered in the differential diagnosis of hypocalcaemia presenting in adulthood, even in the absence of classical morphological features. The possible link between DiGeorge syndrome and malignancy must also be borne in mind when dealing with suspicious mass lesions.

## References

[1] Di George AM, Lischner HW, Dacou C, Arey JB (1967). Absence of the thymus. *The Lancet*.

[2] Wilson DI, Cross IE, Wren C (1994). Minimum prevalence of chromosome 22q11 deletions. *The American Journal of Human Genetics*.

[3] Devriendt K, Fryns JP, Mortier G, Van Thienen MN, Keymolen K (1998). The annual incidence of DiGeorge/velocardiofacial syndrome. *Journal of Medical Genetics*.

[4] Robinson HB (1975). DiGeorge’s or the III-IV pharyngeal pouch syndrome: pathology and a theory of pathogenesis. *Perspectives in Pediatric Pathology*.

[5] Mølsted K, Boers M, Kjär I (2010). The morphology of the sella turcica in velocardiofacial syndrome suggests involvement of a neural crest developmental field. *American Journal of Medical Genetics A*.

[6] Moss E, Wang PP (1995). Characteristic cognitive profile in patients with a 22q11 deletion: verbal IQexceeds nonverbal IQ. *American Journal of Human Genetics*.

[7] Ryan AK, Goodship JA, Wilson DI (1997). Spectrum of clinical features associated with interstitial chromosome 22q11 deletions: a European collaborative study. *Journal of Medical Genetics*.

[8] Sieberer M, Runte I, Wilkening A, Pabst B, Ziegenbein M, Haltenhof H (2006). Spectrum of neuropsychiatric features associated with velocardiofacial syndrome (deletion 22q11.2). *Fortschritte der Neurologie Psychiatrie*.

[9] Murphy KC, Jones LA, Owen MJ (1999). High rates of schizophrenia in adults with velo-cardio-facial syndrome. *Archives of General Psychiatry*.

[10] Kao A, Mariani J, McDonald-McGinn DM (2004). Increased prevalence of unprovoked seizures in patients with a 22q11.2 deletion. *American Journal of Medical Genetics*.

[11] Butts SC (2009). The facial phenotype of the velo-cardio-facial syndrome. *International Journal of Pediatric Otorhinolaryngology*.

[12] Cuneo BF (2001). 22q11.2 deletion syndrome: diGeorge, velocardiofacial, and conotruncal anomaly face syndromes. *Current Opinion in Pediatrics*.

[13] Jawad AF, McDonald-McGinn DM, Zackai E, Sullivan KE (2001). Immunologic features of chromosome 22q11.2 deletion syndrome (DiGeorge syndrome/velocardiofacial syndrome). *Journal of Pediatrics*.

[14] Piliero LM, Sanford AN, McDonald-McGinn DM, Zackai EH, Sullivan KE (2004). T-cell homeostasis in humans with thymic hypoplasia due to chromosome 22q11.2 deletion syndrome. *Blood*.

[15] Chinen J, Rosenblatt HM, Smith EO, Shearer WT, Noroski LM (2003). Long-term assessment of T-cell populations in DiGeorge syndrome. *Journal of Allergy and Clinical Immunology*.

[16] Lavi RF, Kamchaisatian W, Sleasman JW (2006). Thymic output markers indicate immune dysfunction in DiGeorge syndrome. *Journal of Allergy and Clinical Immunology*.

[17] Piliero LM, Sanford AN, McDonald-McGinn DM, Zackai EH, Sullivan KE (2004). T-cell homeostasis in humans with thymic hypoplasia due to chromosome 22q11.2 deletion syndrome. *Blood*.

[18] Pierdominici M, Mazzetta F, Caprini E (2003). Biased T-cell receptor repertoires in patients with chromosome 22q11.2 deletion syndrome (DiGeorge syndrome/velocardiofacial syndrome). *Clinical and Experimental Immunology*.

[19] Gennery AR, Barge D, O’Sullivan JJ, Flood TJ, Abinun M, Cant AJ (2002). Antibody deficiency and autoimmunity in 22q11.2 deletion syndrome. *Archives of Disease in Childhood*.

[20] Finocchi A, Di Cesare S, Romiti ML (2006). Humoral immune responses and CD27^+^ B cells in children with DiGeorge syndrome (22q11.2 deletion syndrome). *Pediatric Allergy and Immunology*.

[21] Smith CA, Driscoll DA, Emanuel BS, McDonald-McGinn DM, Zackai EH, Sullivan KE (1998). Increased prevalence of immunoglobulin a deficiency in patients with the chromosome 22q11.2 deletion syndrome (DiGeorge syndrome/velocardiofacial syndrome). *Clinical and Diagnostic Laboratory Immunology*.

[22] Ryan AK, Goodship JA, Wilson DI (1997). Spectrum of clinical features associated with interstitial chromosome 22q11 deletions: a European collaborative study. *Journal of Medical Genetics*.

[23] Sullivan KE, McDonald-McGinn D, Zackai EH (2002). CD4^+^ CD25^+^ T-cell production in healthy humans and in patients with thymic hypoplasia. *Clinical and Diagnostic Laboratory Immunology*.

[24] Barthlott T, Kassiotis G, Stockinger B (2003). T cell regulation as a side effect of homeostasis and competition. *Journal of Experimental Medicine*.

[25] McDonald-McGinn DM, Reilly A, Wallgren-Pettersson C (2006). Malignancy in chromosome 22q11.2 deletion syndrome (DiGeorge syndrome/Velocardiofacial syndrome). *American Journal of Medical Genetics*.

[26] Kar PS, Ogoe B, Poole R, Meeking D (2005). Di-George syndrome presenting with hypocalcaemia in adulthood: two case reports and a review. *Journal of Clinical Pathology*.

[27] Johnston PC, Donnelly DE, Morrison PJ, Hunter SJ (2009). DiGeorge syndrome presenting as late onset hypocalcaemia in adulthood. *Ulster Medical Journal*.

